# The PARADIGHM (physicians advancing disease knowledge in hypoparathyroidism) registry for patients with chronic hypoparathyroidism: study protocol and interim baseline patient characteristics

**DOI:** 10.1186/s12902-021-00888-2

**Published:** 2021-11-20

**Authors:** Neil Gittoes, Lars Rejnmark, Steven W. Ing, Maria Luisa Brandi, Sigridur Björnsdottir, Stefanie Hahner, Lorenz C. Hofbauer, Pascal Houillier, Aliya A. Khan, Michael A. Levine, Michael Mannstadt, Dolores M. Shoback, Tamara J. Vokes, Pinggao Zhang, Claudio Marelli, John Germak, Bart L. Clarke

**Affiliations:** 1 Centre for Endocrinology, Diabetes and Metabolism (CEDAM), Queen Elizabeth Hospital Edgbaston, 3rd Floor, Heritage Building, Birmingham, B15 2TH UK; 2 Department of Endocrinology and Internal Medicine, Aarhus University Hospital, Aarhus, Palle Juul-Jensens Boulevard 99, 8200 Aarhus N, Denmark; 3 Division of Endocrinology, Diabetes and Metabolism, 547 McCampbell Hall, Ohio State University Wexner Medical Center, 1581 Dodd Drive, Columbus, OH 43210 USA; 4 Endocrinology and Metabolic Diseases, University of Florence, Viale Pieraccini 6, 50139 Florence, Italy; 5 Department of Endocrinology, Metabolism, and Diabetes, Karolinska University Hospital Solna, SE-17176 Stockholm, Sweden; 6 Department of Medicine I Endocrinology, and Diabetology, University Hospital Würzburg, Oberdürrbacher Str. 6, 97080 Würzburg, Germany; 7 Division of Endocrinology, Diabetes, and Bone Diseases, Technische Universität Dresden Medical Center, Fetscherstrasse 74, D-01307 Dresden, Germany; 8 Centre de Recherche des Cordeliers, INSERM, 15 Rue de l’Ecole de Médecine, Sorbonne Université, Université de Paris, Assistance Publique-Hôpitaux de Paris, 75006 Paris, France; 9 Department of Medicine, McMaster University, 3075 Hospital Gate, Oakville, ON L6M 1M1 Canada; 10 Division of Endocrinology and Diabetes, Children’s Hospital of Philadelphia and University of Pennsylvania Perelman School of Medicine, 34th and Civic Center Boulevard, Philadelphia, PA 19104 USA; 11 Endocrine Unit Massachusetts General Hospital and Harvard Medical School, 50 Blossom Street, Boston, MA 02114 USA; 12 Endocrine Research Unit, San Francisco Department of Veterans Affairs Medical Center and University of California, 1700 Owens Street, San Francisco, CA 94158 USA; 13 Section of Endocrinology, University of Chicago Medicine, 5841 South Maryland Avenue, Chicago, IL 60637 USA; 14 Shire Human Genetic Therapies, Inc., a Takeda company, 45 Hayden Ave, Lexington, MA 02421 USA; 15 Takeda Pharmaceuticals International AG, Thurgauerstrasse 130, 8152 Glattpark-Opfikon, Zurich, Switzerland; 16 Division of Endocrinology, Diabetes, Metabolism, and Nutrition, Mayo Clinic, 1st Street SW, Rochester, MN USA

**Keywords:** Chronic hypoparathyroidism, parathyroid hormone, patient registry, quality of life, rhPTH(1-84), symptoms

## Abstract

**Background:**

The PARADIGHM registry of adult and pediatric patients with chronic hypoparathyroidism evaluates the long-term safety and effectiveness of treatment with recombinant human parathyroid hormone, rhPTH(1-84), and describes the clinical disease course under conditions of routine clinical practice. In this first report, we detail the registry protocol and describe the baseline characteristics of two adult patient cohorts from an interim database analysis. One cohort after study entry were prescribed rhPTH(1-84), and the other cohort received conventional therapy of calcium and active vitamin D.

**Methods:**

An observational study of patients with chronic hypoparathyroidism in North America and Europe, collecting data for ≥10 years per patient. Main outcome measures were baseline patient demographics, clinical characteristics, medications, and disease outcome variables of symptoms, biochemical parameters, and health assessments. Baseline is the enrollment assessment for all variables except biochemical measurements in patients treated with rhPTH(1-84); those measurements were the most recent value before the first rhPTH(1-84) dose. Exclusion criteria applied to the analysis of specified outcomes included pediatric patients, patients who initiated rhPTH(1-84) prior to enrollment, and those who received rhPTH(1-34). Clinically implausible biochemical outlier data were excluded.

**Results:**

As of 30 June 2019, data of 737 patients were analyzed from 64 centers; 587 (80%) were women, mean ± SD age 49.1±16.45 years. At enrollment, symptoms reported for patients later prescribed rhPTH(1-84) (n=60) and those who received conventional therapy (n=571), respectively, included fatigue (51.7%, 40.1%), paresthesia (51.7%, 29.6%), muscle twitching (48.3%, 21.9%), and muscle cramping (41.7%, 33.8%). Mean serum total calcium, serum phosphate, creatinine, and estimated glomerular filtration rate were similar between cohorts. Health-related quality of life (HRQoL) 36-item Short Form Health Survey questionnaire scores for those later prescribed rhPTH(1-84) were generally lower than those for patients in the conventional therapy cohort.

**Conclusions:**

At enrollment, based on symptoms and HRQoL, a greater percentage of patients subsequently prescribed rhPTH(1-84) appeared to have an increased burden of disease than those who received conventional therapy despite having normal biochemistry measurements. PARADIGHM will provide valuable real-world insights on the clinical course of hypoparathyroidism in patients treated with rhPTH(1-84) or conventional therapy in routine clinical practice.

**Trial registration:**

EUPAS16927, NCT01922440

**Supplementary Information:**

The online version contains supplementary material available at 10.1186/s12902-021-00888-2.

## Introduction

Hypoparathyroidism is a rare endocrine disorder resulting from undetectable or inappropriately low circulating levels of parathyroid hormone (PTH), which is the principal regulator of calcium and phosphate homeostasis [1, 2]. The main biochemical abnormalities resulting from PTH deficiency are hypocalcemia and hyperphosphatemia [3]. The most common cause of hypoparathyroidism is neck surgery (approximately 75% of cases) [4, 5]. Guidelines from the European Society of Endocrinology; the First International Conference on the Diagnosis, Management, and Treatment of Hypoparathyroidism; and the Canadian-led international working group provide recommendations for the diagnosis, treatment, and monitoring of chronic hypoparathyroidism in adults [1, 4, 6]. Patients with chronic hypoparathyroidism who are treated with conventional therapy consisting of calcium and active vitamin D supplements may still be inadequately controlled and have symptoms and risks of complications and comorbidities [3, 7–11]. In a 13-country patient and caregiver survey, the magnitude of symptom severity experienced by patients whose hypoparathyroidism was not adequately controlled despite receiving conventional therapy was associated with decreased health-related quality of life (HRQoL) and health status assessments and with increased caregiver burden [12]. Evaluation of HRQoL in patients with hypoparathyroidism has proved challenging because of variability of symptoms and differing types of study designs. Many but not all studies found there is a substantial burden of illness that adversely affects HRQoL in patients with chronic hypoparathyroidism [12–21]. A spectrum of symptomatology varying from no symptoms to severe symptoms was reported by patients with chronic hypoparathyroidism; in 36-item Short Form Health Survey questionnaire (SF-36) data transformed on a 0−100 scale (lower scores indicating poorer health states) the scores ranged from 20 to >80 in different domains [12].

The recombinant human parathyroid hormone (1-84), rhPTH(1-84), is full-length PTH that is approved in the United States and Europe as adjunctive treatment for patients with chronic hypoparathyroidism [22, 23]. In Europe, rhPTH(1-84) is indicated as adjunctive treatment for patients with chronic hypoparathyroidism who cannot be adequately controlled with conventional therapy alone. In the United States, rhPTH(1-84) is indicated as an adjunct to calcium and vitamin D to control hypocalcemia and is recommended only for patients who cannot be well controlled with calcium supplements and active vitamin D alone. The safety and efficacy of rhPTH(1-84) in patients with chronic hypoparathyroidism were first shown in short-term placebo-controlled clinical studies in which the need for conventional therapy was reduced, serum calcium levels were maintained, serum phosphate decreased, and events of hypocalcemia were reduced [24, 25]. In longer-term, open-label clinical studies, no new safety concerns were observed, reductions in oral calcium supplements and active vitamin D were achieved, serum calcium remained within target range, serum phosphorus and calcium-phosphate product levels improved, and urinary calcium excretion normalized [26–29].

Observational studies complement findings from interventional clinical trials and can provide real-world information on long-term outcomes of safety, effectiveness, and HRQoL across a broad population [30–32]. Patient registries, in particular for rare diseases, can obtain natural disease or treatment response data over the long-term that would otherwise be limited if performed by individual centers [31].

The multinational PARADIGHM (physicians advancing disease knowledge in hypoparathyroidism) registry (NCT01922440, EUPAS register number: EUPAS16927) is an opportunity to obtain valuable real-world data beyond those reported for clinical trials in patients with chronic hypoparathyroidism by providing long-term data for the treatment of patients with chronic hypoparathyroidism with rhPTH(1-84). PARADIGHM also records data that will allow for the assessment of the overall disease course in patients under conditions of routine clinical practice. Herein we describe the protocol for PARADIGHM and report baseline demographic and clinical characteristics for the current participants.

## Registry protocol

### Design

PARADIGHM is a prospective, observational registry designed to collect long-term data on patients with chronic hypoparathyroidism, whether they are receiving rhPTH(1-84) or conventional therapy, or both, under conditions of routine clinical practice. The registry data collection began in 2013, and enrollment is planned to close when an adequate number of patients have been enrolled to meet the study objectives. The original registry protocol was subsequently amended after rhPTH(1-84) regulatory approval to include the designation as a non-interventional post-authorization safety study and to fulfill the post-marketing commitment request from the European Medicines Agency to the sponsor Shire, now a Takeda company. Data collection will continue for a minimum of ten years of follow-up for each patient; a final report is planned for 2035 or at the end of the product life cycle (ie, permanent cessation of release into the market/distribution chain) unless agreed otherwise with regulatory agencies.

### Objectives

The protocol-defined primary objective of PARADIGHM is to characterize and describe the long-term safety and effectiveness of rhPTH(1-84) in patients with chronic hypoparathyroidism under conditions of routine clinical practice. The secondary objective is to characterize and describe the course of chronic hypoparathyroidism, including overall health status, patient-reported outcomes for symptoms of hypoparathyroidism, and healthcare resource utilization in patients treated or not treated with rhPTH(1-84) under conditions of routine clinical practice.

### Population

The registry aims to enroll at least 900 patients with chronic hypoparathyroidism, including at least 300 patients receiving treatment with rhPTH(1-84) and at least 600 patients receiving conventional therapy alone (that includes calcium supplements and active vitamin D). All decisions regarding the management of chronic hypoparathyroidism for each patient are determined by the treating physician; no medications or recommendations are provided through participation in the registry. All adult patients (aged ≥18 years) and pediatric patients (aged <18 years) with a diagnosis of chronic hypoparathyroidism (ie, hypoparathyroidism with a duration of ≥6 months) receiving rhPTH(1-84) or conventional therapy are eligible for participation in the registry. Inclusion and exclusion criteria are listed in Table 1. The patient, the parent(s) of a pediatric patient, or a legally authorized representative is required to provide written informed consent. Patients may withdraw at any time for any reason without prejudice to their current or subsequent care. Patients who withdraw from the registry will be allowed to reenter, and any available data from the period the patient was not enrolled will be entered into the electronic case report form (eCRF). The study protocol is reviewed and approved by the Institutional Review Board (IRB)/Independent Ethics Committee/Research Ethics Board at each participating site or a centralized IRB service provider.

**Table 1:** Criteria for registry participants

Inclusion	
• Patients with a diagnosis of chronic hypoparathyroidism and with a duration of ≥6 months	
– Adult patients aged ≥18 years who are receiving conventional therapy, conventional therapy plus rhPTH(1-84), or rhPTH(1-84) alone	
– Pediatric patients aged <18 years who are receiving conventional therapy, conventional therapy plus rhPTH(1-84), or rhPTH(1-84) alone	
Exclusion	
• Patients or legally authorized representatives unable to provide informed consent	
• Patients using rhPTH(1-34), or used rhPTH(1-34) for >2 years and in the past 3 months	
• Patients currently enrolled in an interventional clinical study whether or not the study is related to hypoparathyroidism^a^	
• History of hypoparathyroidism resulting from a known activating mutation in the *CASR* gene	
• History of hypoparathyroidism resulting from impaired responsiveness to PTH (pseudohypoparathyroidism)	

*PTH* parathyroid hormone, *rhPTH(1-34)* recombinant human parathyroid hormone (1-34), *rhPTH(1-84)* recombinant human parathyroid hormone (1-84)

^a^Does not apply to those enrolled in other observational registries. The criteria were added during amendments to the original protocol to account for the inclusion of registry participants treated with rhPTH(1-84) prior to registry enrollment

### Data collection

The specific data to be entered into the registry database using the eCRFs at baseline and during subsequent follow-up clinic visits are listed in Table 2. Baseline is defined as the assessment at enrollment (visit 1) for all outcome variables except biochemical laboratory measurements in patients receiving treatment with rhPTH(1-84). The baseline for biochemical laboratory measurements was the most recent value before the first dose in patients receiving treatment with rhPTH(1-84). Patient data are to be entered into the registry database at intervals of at least every six months, except for adverse events (AEs), which are collected on a continuous basis. The registry protocol AE definition is any pathologic, noxious, or unintended change in function indicated by changes in physical signs, symptoms, or laboratory evaluations. These include exacerbation of a preexisting condition and whether the AE is considered by the investigator as related to treatment with rhPTH(1-84) or not. Classification as an AE was at the investigator discretion after the patient signed the informed consent form, but AEs should not have been historical events prior to enrollment. AEs will be coded using the *Medical Dictionary for Regulatory Activities* (version 19.1 or later). The registry protocol serious AE (SAE) definition is any event that is life threatening or leads to death, requires inpatient hospitalization or prolongation of hospitalization, results in persistent or significant disability or incapacity, is a congenital abnormality or birth defect, or any other important medical event whether the SAE is considered by the investigator as related to treatment with rhPTH(1-84) or not.

**Table 2:** Data collection schedule

Parameter	Baseline visit	Follow-up visit
Inclusion/exclusion criteria	×	
Informed consent, medical records release, and contact order form	×	
Demographics^a^	×	
Other study participation	×	×
Hypoparathyroidism etiology (primary cause)^b^	×	
Family history	×	
Medical history/condition summary	×	
Height and weight	×	×
Pregnancy	×	×
Clinical laboratory evaluations	×	×
Other medical procedures^c^	×	×
Management of chronic hypoparathyroidism	×	×
rhPTH(1-84) dosing information^d^	×	×
Historical PTH dosing information^e^	×	
Prior and concomitant medications, including over-the-counter medications	×	×
Outcome evaluations (socioeconomic status and social history)	×	
Outcome evaluations (Hypoparathyroidism Symptom Diary, WPAI:SHP, SF-36, SF-10)	×^f^	×
Other questionnaires (signs and symptoms, hospitalization annual form)	×	×
Vital status database searches		×^g^
Adverse events	×^f,h^	×
Patient discontinuation		×

*PTH* parathyroid hormone, *rhPTH(1-84)* recombinant human parathyroid hormone (1-84), *SF-10* 10-Item Short Form Health Survey questionnaire for pediatric patients, *SF-36* 36-Item Short Form Health Survey questionnaire for adults, *WPAI:SHP* Work Productivity and Activity Impairment Specific Health Problem

^a^For example, month and year of birth, age

^b^Genetic mutation, surgery-induced, autoimmune, radiation, idiopathic, other

^c^For example, dual-energy x-ray absorptiometry, imaging, bone biopsies

^d^Refers to the use of rhPTH(1-84) (Natpar®/Natpara®)

^e^Refers to the use of rhPTH(1-84) (Preotact®) or rhPTH(1-34) (Forteo®)

^f^Discrepancy from November 2017 protocol to be corrected in future amendments

^g^Search is only performed if patient is a US patient lost to follow-up or reported deceased

^h^Classification was at investigator discretion after the signing of the informed consent form (ie, adverse events should not be historical events before enrollment)

### Outcome variables

The primary outcome variables are AEs; clinical laboratory test results; biochemical laboratory test results for 24-hour urine calcium, serum total calcium, albumin-corrected serum calcium, magnesium and phosphate (measured as inorganic phosphate) serum levels, and 25[OH]D; renal function assessments, including serum creatinine, creatinine-based estimated glomerular filtration rate (eGFR; analyzed in this report using the Chronic Kidney Disease Epidemiology Collaboration equation) [33], 24-hour urine calcium, and 24-hour urine protein; renal calcification (eg nephrolithiasis, nephrocalcinosis); visits to an emergency department (ED) or hospitalization for renal events; presence of cataract; sites and numbers of bone fractures; and cardiovascular events.

The secondary outcome variables include HRQoL evaluated using the Short Form Health Survey questionnaires (SF-36 for adult patients, SF-10 for pediatric patients), disease-specific patient-reported outcomes using the Hypoparathyroidism Symptom Diary [34, 35], chronic hypoparathyroidism-related visits to an ED or hospitalization, or other reasons for visits to an ED or hospitalization. In addition, the impact on daily life will be assessed using the Work Productivity and Activity Impairment Specific Health Problem questionnaire [36, 37].

### Statistical analysis

No formal statistical testing is preplanned for registry data analyses. In general, categorical outcome variables are summarized as number of patients, percentage, and 95% CI of patients in each category, and continuous outcome variables are summarized using descriptive statistics as number of patients, mean, SD, 95% CI, and median (range).

## Interim registry database analysis: patient demographics and baseline characteristics

This manuscript reports on the baseline patient demographic and clinical characteristics data collected for the overall analysis population and two adult patient cohorts predefined in the study design protocol: 1) patients prescribed rhPTH(1-84) after enrollment, and 2) patients receiving conventional therapy alone. Patients treated with rhPTH(1-34) were excluded, and patients who initiated treatment with rhPTH(1-84) prior to enrollment were excluded from specific analyses where this treatment could influence data reported at the time of enrollment (signs and symptoms of hypoparathyroidism reported within six months of enrollment; doctors’ office visits, ED visits, and overnight hospital admissions due to hypoparathyroidism; and HRQoL SF-36 questionnaire). Analysis exclusions are noted within each relevant Table footer. In addition, biochemical parameters excluded patients with outlier data that after review were considered clinically implausible for patients with serum total calcium <0.88 mmol/L, >5.00 mmol/L (<3.5 mg/dL, >20.0 mg/dL); serum phosphate <0.32 mmol/L, >2.58 mmol/L (<1.0 mg/dL, >8.0 mg/dL); serum creatinine <17.68 μmol/L, >884.00 μmol/L (<0.2 mg/dL, >10.0 mg/dL); or eGFR with negative values or values >200.0 mL/min/1.73 m^2^.

The baseline data reported herein is for the following parameters: patient demographics; clinical characteristics; concomitant medications; disease-reported signs and symptoms; hypoparathyroidism-relevant biochemical parameters measured as part of routine clinical practice for serum total calcium, serum phosphate, serum creatinine and eGFR; the number of doctors’ office visits, ED visits, and overnight hospital admissions in the 12 months before baseline; and HRQoL evaluated SF-36 questionnaires for adult patients.

As of 30 June 2019, the analysis population consisted of 737 patients (mean ± SD age, 49.1±16.45 years) from 64 centers in Europe and North America. The largest number of patients enrolled at a single center was 79, two centers had enrolled 47 patients, four centers had enrolled between 20 and 40 patients, and 57 centers had enrolled <20 patients. The number of patients enrolled at each center is shown in Additional file 1. The study protocol was approved by each participating site via Independent Ethics Committees, Research Ethics Boards, or Institutional Review Boards (IRBs). For approvals by IRBs, either an institution-specific (27 US sites) or a centralized service provider who was able to review and approve for multiple sites (1 UK, 4 Swedish, and 19 US sites) were used. The Institutional Review Board of the Mayo Clinic, Rochester, Minnesota, USA approved the protocol for use at that institution.

The baseline patient demographics and clinical characteristics of the 737 participants are shown in Table 3. Most physicians treating patients with chronic hypoparathyroidism were endocrinologists (n=660, 89.6%). In adult patients, the most frequently used concomitant medications are shown in Table 4. Active vitamin D and analogs were prescribed for 90.2%, calcium for 80.7%, and thyroid hormones for 73.3% of adult patients. Antidepressants were prescribed more frequently for patients who were subsequently prescribed rhPTH(1-84) than in the conventional therapy cohort.

**Table 3:** Baseline patient demographics and clinical characteristics

Parameter	rhPTH(1-84) (***n***=134)	Conventional Therapy (***n***=603)	Analysis Population^a^ (***N***=737)
Age, mean ± SD, y	47.9±14.22	49.3±16.91	49.1±16.45
Age, category, n (%), y			
0–17	3 (2.2)	24 (4.0)	27 (3.7)
18–39	34 (25.4)	135 (22.4)	169 (22.9)
40–64	81 (60.4)	336 (55.7)	417 (56.6)
≥65	16 (11.9)	108 (17.9)	124 (16.8)
Women, n (%)	115 (85.8)	472 (78.3)	587 (79.6)
Race, white, n (%)	112 (83.6)	508 (84.2)	620 (84.1)
BMI, mean ± SD, y			
<18^b^	17.2±0.46	19.6±6.07	19.3±5.73
≥18^c^	31.1±9.48	29.7±7.32	30.0±7.72
Primary cause of hypoparathyroidism, n (%)			
Surgery	85 (63.4)	462 (76.6)	547 (74.2)
Idiopathic	12 (9.0)	46 (7.6)	58 (7.9)
Genetic and autoimmune	12 (9.0)	44 (7.3)	56 (7.6)
Radiation	0	1 (0.2)	1 (0.1)
Unknown/other/missing	25 (18.7)	50 (8.3)	75 (10.2)
Primary cause for thyroid surgery			
Thyroid cancer, n (%)	37 (56.1)	244 (60.7)	281 (60.0)
Duration of hypoparathyroidism, mean ± SD, y^d^	9.0±10.27	10.3±11.53	10.1±11.34

*BMI* body mass index, *rhPTH(1-84)* recombinant human parathyroid hormone (1-84)

^a^Baseline is defined as the assessment at enrollment (visit 1); 737 patients as of 30 June 2019

^b^rhPTH(1-84) *n*=3, conventional therapy *n*=22

^c^rhPTH(1-84) *n*=97, conventional therapy *n*=490

^d^rhPTH(1-84) *n*=110, conventional therapy *n*=563

**Table 4:** Baseline concomitant medications used by ≥10% of adult patients^a^

Medication, n (%)^b^	rhPTH(1-84) (***n***=125)	Conventional therapy (***n***=571)	Analysis population^c^ (***N***=696)
Vitamin D and analogs	105 (84.0)	523 (91.6)	628 (90.2)
Calcitriol	86 (68.8)	430 (75.3)	516 (74.1)
Native vitamin D, including cholecalciferol and ergocalciferol	75 (60.0)	255 (44.7)	330 (47.4)
Alfacalcidol	1 (0.8)	55 (9.6)	56 (8.0)
Calcium	96 (76.8)	466 (81.6)	562 (80.7)
Calcium carbonate	62 (49.6)	333 (58.3)	395 (56.8)
Calcium citrate	46 (36.8)	148 (25.9)	194 (27.9)
Calcium, not otherwise specified	9 (7.2)	39 (6.8)	48 (6.9)
Thyroid hormone	86 (68.8)	424 (74.3)	510 (73.3)
Levothyroxine	79 (63.2)	417 (73.0)	496 (71.3)
Magnesium	46 (36.8)	140 (24.5)	186 (26.7)
Proton pump inhibitors	26 (20.8)	127 (22.2)	153 (22.0)
Benzodiazepine derivatives	26 (20.8)	74 (13.0)	100 (14.4)
HMG-CoA reductase inhibitors	16 (12.8)	115 (20.1)	131 (18.8)
Potassium	25 (20.0)	66 (11.6)	91 (13.1)
Combinations of vitamins	23 (18.4)	91 (15.9)	114 (16.4)
Antidepressants	23 (18.4)	56 (9.8)	79 (11.4)
Selective serotonin reuptake inhibitors	22 (17.6)	70 (12.3)	92 (13.2)
Platelet aggregation inhibitors^d^	10 (8.0)	92 (16.1)	102 (14.7)
Glucocorticoids	17 (13.6)	91 (15.9)	108 (15.5)
Thiazides, hydrochlorothiazide	16 (12.8)	76 (13.3)	92 (13.2)
Beta-blocking agents, selective	15 (12.0)	67 (11.7)	82 (11.8)

*HMG-CoA reductase* 3-hydroxy-3-methyl-glutaryl-coenzyme A reductase, *rhPTH(1-84)* recombinant human parathyroid hormone (1-84)

^a^Drug class cutoff based on analysis population

^b^Medication coded using the World Health Organization Drug Dictionary

^c^Baseline is defined as the assessment at enrollment (visit 1); pediatric patients (*n*=27) and those who received rhPTH(1-34) (*n*=14) were excluded from the analysis

^d^Excludes heparin

The most commonly reported signs and symptoms of hypoparathyroidism in adult patients within the previous six months of enrollment are shown in Table 5. At enrollment, muscle twitching and muscle cramping were reported in 48.3% and 41.7% of patients who were subsequently prescribed rhPTH(1-84) (*n*=60) and 21.9% and 33.8% of patients who received conventional therapy (*n*=571), respectively. Fatigue and anxiety were reported in 51.7% and 26.7% of patients subsequently prescribed rhPTH(1-84) (*n*=60) and 40.1% and 20.7% of patients who received conventional therapy (*n*=571), respectively.

**Table 5:** Common symptoms reported within 6 months before enrollment (≥15% of adult patients in either cohort)

Parameter, n (%)	rhPTH(1-84) (***n***=60)	Conventional therapy (***n***=571)	Analysis population^a^ (***N***=631)
Fatigue	31 (51.7)	229 (40.1)	260 (41.2)
Paresthesia	31 (51.7)	169 (29.6)	200 (31.7)
Muscle twitching	29 (48.3)	125 (21.9)	154 (24.4)
Muscle cramping	25 (41.7)	193 (33.8)	218 (34.5)
Headache	20 (33.3)	105 (18.4)	125 (19.8)
Muscle pain	17 (28.3)	112 (19.6)	129 (20.4)
Brain fog	17 (28.3)	95 (16.6)	112 (17.7)
Muscle weakness	17 (28.3)	88 (15.4)	105 (16.6)
Tetany	17 (28.3)	71 (12.4)	88 (13.9)
Joint pain	16 (26.7)	138 (24.2)	154 (24.4)
Anxiety	16 (26.7)	118 (20.7)	134 (21.2)
Weakness in extremities	12 (20.0)	83 (14.5)	95 (15.1)
Pain in extremities	11 (18.3)	83 (14.5)	94 (14.9)
Bone pain	11 (18.3)	76 (13.3)	87 (13.8)
Constipation	11 (18.3)	72 (12.6)	83 (13.2)
Nausea	11 (18.3)	52 (9.1)	63 (10.0)
Back pain	9 (15.0)	106 (18.6)	115 (18.2)

*rhPTH(1-84)* recombinant human parathyroid hormone (1-84)

^a^The assessment at enrollment (visit 1) was defined as baseline; pediatric patients (*n*=27), those who received rhPTH(1-34) (*n*=14), and those who initiated rhPTH(1-84) before enrollment (*n*=65) were excluded from the analysis

Hypoparathyroidism-relevant biochemical parameters measured as part of routine clinical practice included serum total calcium, serum phosphate, serum creatinine, and eGFR and were similar in adult patients in both groups (Table 6). Adult patients who were subsequently prescribed rhPTH(1-84) showed a trend for higher numbers of doctors’ office visits and ED visits in the 12 months before baseline versus those who received conventional therapy (Table 7). In addition, 45.0% of patients who were later prescribed rhPTH(1-84) and 14.5% of patients who received conventional therapy had ≥1 overnight hospital admission over the same time frame (Table 7).

**Table 6:** Baseline biochemical parameters in adult patients

Parameter	rhPTH(1-84) (***n***=125)		Conventional therapy (***n***=571)		Analysis population^a^ (***N***=696)	
	n	Value	n	Value	n	Value
Total serum calcium, mmol/L						
Mean ± SD	83	2.2±0.34	489	2.2±0.21	572	2.2±0.24
Median (range)		2.2 (1.30–3.53)		2.2 (1.15–3.15)		2.2 (1.15–3.53)
Serum phosphate^b^, mmol/L						
Mean ± SD	53	1.3±0.27	280	1.4±0.26	333	1.4±0.26
Median (range)		1.3 (0.8–1.9)		1.3 (0.7–2.1)		1.3 (0.7–2.1)
Serum creatinine, μmol/L						
Mean ± SD	69	86.9±55.93	452	84.1±30.80	521	84.5±35.11
Median (range)		74.3 (38.9–479.1)		78.3 (26.5–284.6)		77.8 (26.5–479.1)
eGFR, mL/min/1.73 m^2^						
Mean ± SD	69	83.2±23.73	454	80.2±24.34	523	80.6±24.25
Median (range)		84.9 (8.2–128.5)		81.1 (0.5–162.5)		81.4 (0.5–162.5)

*eGFR* estimated glomerular filtration rate, *rhPTH(1-84)* recombinant human parathyroid hormone (1-84)

^a^Baseline is defined as the assessment at enrollment (visit 1). The baseline measurement was the most recent value before the first dose in patients receiving treatment with rhPTH(1-84); pediatric patients (*n*=27), those who received rhPTH(1-34) (n=14), and those with outlier data for total serum calcium <0.88 mmol/L, >5.00 mmol/L (<3.5 mg/dL, >20.0 mg/dL); serum phosphate <0.32 mmol/L, >2.58 mmol/L (<1.0 mg/dL, >8.0 mg/dL); serum creatinine <17.68 μmol/L, >884.00 μmol/L (<0.2 mg/dL, >10.0 mg/dL); eGFR no lower limit/except negative values excluded, >200.0 mL/min/1.73 m^2^, were excluded from the analysis

^b^Inorganic phosphate was measured

**Table 7:** Doctors’ office visits, emergency department visits, and overnight hospital admissions due to hypoparathyroidism at baseline^a^

Parameter, n (%)	rhPTH(1-84) (***n***=60)	Conventional therapy (***n***=571)	Analysis population^b^ (***N***=631)
Number of doctors’ office visits			
0	8 (13.3)	123 (21.5)	131 (20.8)
1	5 (8.3)	82 (14.4)	87 (13.8)
2–3	20 (33.3)	148 (25.9)	168 (26.6)
4–5	6 (10.0)	42 (7.4)	48 (7.6)
6–7	3 (5.0)	18 (3.2)	21 (3.3)
8–9	7 (11.7)	15 (2.6)	22 (3.5)
10–12	3 (5.0)	9 (1.6)	12 (1.9)
13–15	1 (1.7)	2 (0.4)	3 (0.5)
≥16	2 (3.3)	6 (1.1)	8 (1.3)
Missing	5 (8.3)	126 (22.1)	131 (20.8)
Number of emergency department visits			
0	11 (18.3)	86 (15.1)	97 (15.4)
1	15 (25.0)	41 (7.2)	56 (8.9)
2–3	5 (8.3)	25 (4.4)	30 (4.8)
4–5	3 (5.0)	6 (1.1)	9 (1.4)
6–7	3 (5.0)	8 (1.4)	11 (1.7)
8–9	0 (0.0)	0 (0.0)	0 (0.0)
10–12	1 (1.7)	1 (0.2)	2 (0.3)
13–15	0 (0.0)	1 (0.2)	1 (0.2)
≥16	0 (0.0)	0 (0.0)	0 (0.0)
Missing	22 (36.7)	403 (70.6)	425 (67.4)
≥1 overnight hospital admission	27 (45.0)	83 (14.5)	110 (17.4)

*rhPTH(1-84)* recombinant human parathyroid hormone (1-84)

^a^Data are events reported in adult patients that occurred within 12 months before baseline

^b^Baseline is defined as the assessment at enrollment (visit 1); pediatric patients (*n*=27), those who received rhPTH(1-34) (*n*=14), and those who initiated rhPTH(1-84) before enrollment (*n*=65) were excluded from the analysis

At baseline, the mean ± SD SF-36 physical and mental component summary scores were 42±10.3 and 42±12.8 for patients in the subsequently treated rhPTH(1-84) cohort (n=60) and 46±10.4 and 48±10.8 for patients in the conventional therapy cohort (n=507), respectively. Reported outcome scores across individual SF-36 domains are illustrated in Table 8, which showed that scores at enrollment for patients subsequently treated with rhPTH(1-84) after enrollment trended lower in all domains than those who received conventional therapy.

**Table 8:** Baseline-reported outcome scores for SF-36 domains in adult patients

SF-36 Domain	rhPTH(1-84) (***n***=63)		Conventional therapy (***n***=579)		Analysis population^a^ (***N***=642)	
	n	Value	n	Value	n	Value
Physical functioning	62	43±11.6	525	47±10.1	587	47±10.3
Bodily pain	62	44±10.4	530	47±10.8	592	47±10.8
Role limitation – physical health	62	42±11.4	527	46±11.0	589	46±11.1
Role limitation – emotional problems	62	42±14.4	530	47±11.1	592	47±11.6
General health	61	41±10.4	528	46±11.5	589	45±11.5
Mental health	61	45±12.0	529	49±10.2	590	49±10.5
Social functioning	61	42±11.1	530	47±10.9	591	46±11.0
Vitality	61	42±11.5	529	47±11.1	590	47±11.2

All values are presented as mean ± SD

*rhPTH(1-84)* recombinant human parathyroid hormone (1-84), *SF-36* 36-Item Short Form Health Survey questionnaire for adults

^a^Baseline is defined as the assessment at enrollment (visit 1); pediatric patients (*n*=27) and those who initiated rhPTH(1-84) before enrollment (*n*=68) were excluded from the analysis

## Registry and interim analysis limitations

Information gathered from the large number of patients with chronic hypoparathyroidism enrolled in PARADIGHM will provide extensive outcome data relevant to routine clinical practice in patients treated with conventional therapy or rhPTH(1-84). However, because of the observational study design, there may be unobserved and/or inaccurate data that may limit generalized interpretation of the study outcomes. Because the PARADIGHM registry is ongoing, activity continues within participating centers to reconcile all missing data. In addition, no formal statistical comparisons between cohorts were planned, and the registry is not powered for hypothesis testing to compare outcome variables between the two cohorts. Furthermore, relatively few patients with nonsurgical etiologies for chronic hypoparathyroidism have enrolled in PARADIGHM; as a result, information collected in the registry may be most relevant to patients with post-surgical hypoparathyroidism. Finally, because of the low prevalence of this disease, some participating clinical centers have enrolled only one or a few patients into the registry. The registry may be representative of patient populations in Europe and North America, which have a large number of centers with enrolled patients, but a broader demographic and regional representation could be achieved with additional global (ie, Asia, Australia, Africa, and South America) participation of centers treating patients with chronic hypoparathyroidism.

## Discussion

Because hypoparathyroidism is a rare disease, this multinational registry will enroll more patients with chronic hypoparathyroidism than is feasible for any single center or clinical trial. The design of the study protocol enables the collection of robust real-world data and informative disease-relevant parameters. It is expected that findings from PARADIGHM will increase the understanding of the course of hypoparathyroidism, provide insight into treatment effects in the patient populations, and stimulate further international effort and cooperation that may improve current clinical practices.

In this first report of the PARADIGHM registry, in addition to describing the protocol, we analyzed the baseline characteristics of the currently enrolled patient population. Despite serum calcium levels being maintained within the normal range, signs and symptoms of hypocalcemia, including paresthesia and muscle twitching, can persist in patients with chronic hypoparathyroidism that contribute toward the burden of illness, suggesting that the disease is not adequately controlled in these patients [9, 38]. At enrollment, the cohort of patients who were later prescribed rhPTH(1-84) showed a trend for increased reporting of symptoms related to chronic hypoparathyroidism, including paresthesia and muscle twitching, versus patients who received conventional therapy alone. Chronic hypoparathyroidism is associated with a range of physical, emotional, and mental health symptoms that impact the quality of life of patients, and which also impacts their caregivers [12, 14, 39]. At baseline, use of antidepressants was numerically higher in patients who were later prescribed rhPTH(1-84) than in the conventional therapy cohort, and SF-36 scores across all domains were numerically lower for patients who were later prescribed rhPTH(1-84) than for patients who received conventional therapy. These data suggest that at baseline, patients enrolled in the rhPTH(1-84) group may have had a higher burden of symptoms than those in the conventional therapy group. The data presented in this publication predate any interruption of data collection due to the recall of rhPTH(1-84) in the United States. Any patient who would discontinue rhPTH(1-84) treatment for any reason, including temporary product recall, is eligible for continued follow-up in the registry. Any change in therapy, from rhPTH(1-84) to conventional therapy or from conventional therapy to rhPTH(1-84), for any reason and at any time during the study is consistent with the registry protocol design and would be accounted for in data analysis according to the statistical analysis plan. Continuing assessment of biochemical and other parameters related to health status over the longer term will allow any differences in disease progression to be evaluated.

Information collected in PARADIGHM for patients with hypoparathyroidism encompasses areas of specific interest that are also included in other real-world registries, but there are a number of features that differentiate PARADIGHM from other national registries. The Danish National Patient Registry (DNPR) is a population-based administrative registry that has collected data from all Danish hospitals with complete coverage since 1978 [40]. An aim of the DNPR registry is to monitor the frequency of various diseases and treatments, whereas PARADIGHM collects detailed information on biochemical parameters and overall health status of patients with chronic hypoparathyroidism. The DNPR includes administrative data, diagnoses, treatment, and examinations that provide a sampling resource for longitudinal population-based clinical research. Patients with hypoparathyroidism in PARADIGHM had similar characteristics and etiology to those in the DNPR and Canadian National Hypoparathyroidism Registry (CNHR) that was formed in 2014. Although patient characteristics in PARADIGHM overall were similar to those in the CNHR, 81% of patients were reported to be receiving calcium and 74% were receiving calcitriol in PARADIGHM compared with 100% receiving calcium supplements and 82.2% receiving calcitriol in the CNHR [41].

Despite the noted limitations, the real-world data gathered by PARADIGHM integrate patient demographics, clinical characteristics, biochemical data, and comorbidity data with symptom burden and impact of chronic hypoparathyroidism on quality of life that goes beyond that of other registries and clinical studies [12, 24–28, 42–45]. The added value in the data will also come from the intended long follow-up period and data that will be captured for both adult and pediatric patients, including the opportunity to collect data for pediatric patients as they transition to adulthood.

In summary, PARADIGHM is expected to become a valued information resource about the clinical course of hypoparathyroidism, whether patients are treated with conventional therapy or rhPTH(1-84). PARADIGHM will also provide valuable longitudinal data on the safety and effectiveness of rhPTH(1-84) and may assist clinicians in the future management of patients with chronic hypoparathyroidism.

## Supplementary Information

**Additional file 1: Figure S1.** Number of patients in the analysis population of 737 patients enrolled at each of the 64 participating centers as of 30 June 2019.

## Data Availability

The dataset supporting the conclusions of this article are included within the article. Restrictions apply to the availability of data generated or analyzed during this study to preserve patient confidentiality or because they were used under license. The corresponding author will on request detail the restrictions and any conditions under which access to some data may be provided.

## Abbreviations

AE: adverse event
CNHR: Canadian National Hypoparathyroidism Registry
DNPR: Danish National Patient Registry
eCRF: electronic case report form
ED: emergency department
eGFR: estimated glomerular filtration rate
HRQoL: health-related quality of life
PARADIGHM: Physicians Advancing Disease Knowledge in Hypoparathyroidism
PTH: parathyroid hormone
rhPTH(1-84): recombinant human parathyroid hormone (1-84)
SF-10: 10-item Short Form Health Survey questionnaire for pediatric patients
SF-36: 36-item Short Form Health Survey questionnaire for adults
WPAI:SHP: Work Productivity and Activity Impairment Specific Health Problem
1,25(OH)_2_D: 1,25-dihydroxyvitamin D
25(OH)D: 25-hydroxyvitamin D
