# Supplementary material for: The PARADIGHM (physicians advancing disease knowledge in hypoparathyroidism) registry for patients with chronic hypoparathyroidism: study protocol and interim baseline patient characteristics
Source: BMC Endocr Disord. 2021 Nov 20;21:232. doi: 10.1186/s12902-021-00888-2 (PMC8606089; doi:10.1186/s12902-021-00888-2)

**The PARADIGHM (physicians advancing disease knowledge in hypoparathyroidism) registry for patients with chronic hypoparathyroidism: study protocol and interim baseline patient characteristics**

**Authors:** Neil Gittoes,^1^ Lars Rejnmark,^2^ Steven W. Ing,^3^ Maria Luisa Brandi,^4^ Sigridur Björnsdottir,^5^ Stefanie Hahner,^6^ Lorenz C. Hofbauer,^7^ Pascal Houillier,^8^ Aliya A. Khan,^9^ Michael A. Levine,^10^ Michael Mannstadt,^11^ Dolores M. Shoback,^12^ Tamara J. Vokes,^13^ Pinggao Zhang,^14^ Claudio Marelli,^15^ John Germak,^14^ Bart L. Clarke^16^

**Affiliations:** ^1^Centre for Endocrinology, Diabetes and Metabolism, Heritage Building, Queen Elizabeth Hospital Edgbaston, Birmingham, B15 2TH, UK; ^2^Department of Endocrinology and Internal Medicine, Aarhus University Hospital, Aarhus, Palle Juul-Jensens Boulevard 99, 8200 Aarhus N, Denmark; ^3^Division of Endocrinology, Diabetes and Metabolism, 547 McCampbell Hall, Ohio State University Wexner Medical Center, 1581 Dodd Drive, Columbus, OH 43210, USA; ^4^Endocrinology and Metabolic Diseases, University of Florence, Viale Pieraccini 6, Florence 50139, Italy; ^5^Department of Endocrinology, Metabolism, and Diabetes, Karolinska University Hospital Solna, Stockholm SE-17176, Sweden; ^6^Department of Medicine I Endocrinology, and Diabetology, University Hospital Würzburg, Oberdürrbacher Str. 6, Würzburg 97080, Germany; ^7^Division of Endocrinology, Diabetes, and Bone Diseases, Technische Universität Dresden Medical Center, Fetscherstrasse 74, Dresden D-01307, Germany; ^8^Centre de Recherche des Cordeliers, INSERM, 15 Rue de l'Ecole de Médecine, Sorbonne Université, Université de Paris, Assistance Publique-Hôpitaux de Paris, 75006 Paris, France; ^9^Department of Medicine, McMaster University, 3075 Hospital Gate, Oakville, ON L6M 1M1, Canada; ^10^Division of Endocrinology and Diabetes, Children’s Hospital of Philadelphia and University of Pennsylvania Perelman School of Medicine, 34th and Civic Center Boulevard, Philadelphia, PA 19104, USA; ^11^Endocrine Unit Massachusetts General Hospital and Harvard Medical School, 50 Blossom Street, Boston, MA 02114, USA; ^12^Endocrine Research Unit, San Francisco Department of Veterans Affairs Medical Center and University of California, 1700 Owens Street, San Francisco, CA 94158, USA; ^13^Section of Endocrinology, University of Chicago Medicine, 5841 South Maryland Avenue, Chicago, IL 60637, USA; ^14^Shire Human Genetic Therapies, Inc., a Takeda company, 45 Hayden Ave, Lexington, MA 02421, USA; ^15^Takeda Pharmaceuticals International AG, Thurgauerstrasse 130, 8152 Glattpark-Opfikon, Zurich, Switzerland; ^16^Division of Endocrinology, Diabetes, Metabolism, and Nutrition, Mayo Clinic, 1st Street SW, Rochester, MN, USA

**Corresponding author contact information:** Neil Gittoes, Centre for Endocrinology, Diabetes and Metabolism (CEDAM), 3rd Floor, Heritage Building; Queen Elizabeth Hospital, Edgbaston, Birmingham, B15 2TH, UK. Neil.Gittoes@uhb.nhs.uk. +44 (0) 121 371 6934

**Figure**. Number of patients in the analysis population of 737 patients enrolled at each of the 64 participating centers as of 30 June 2019.


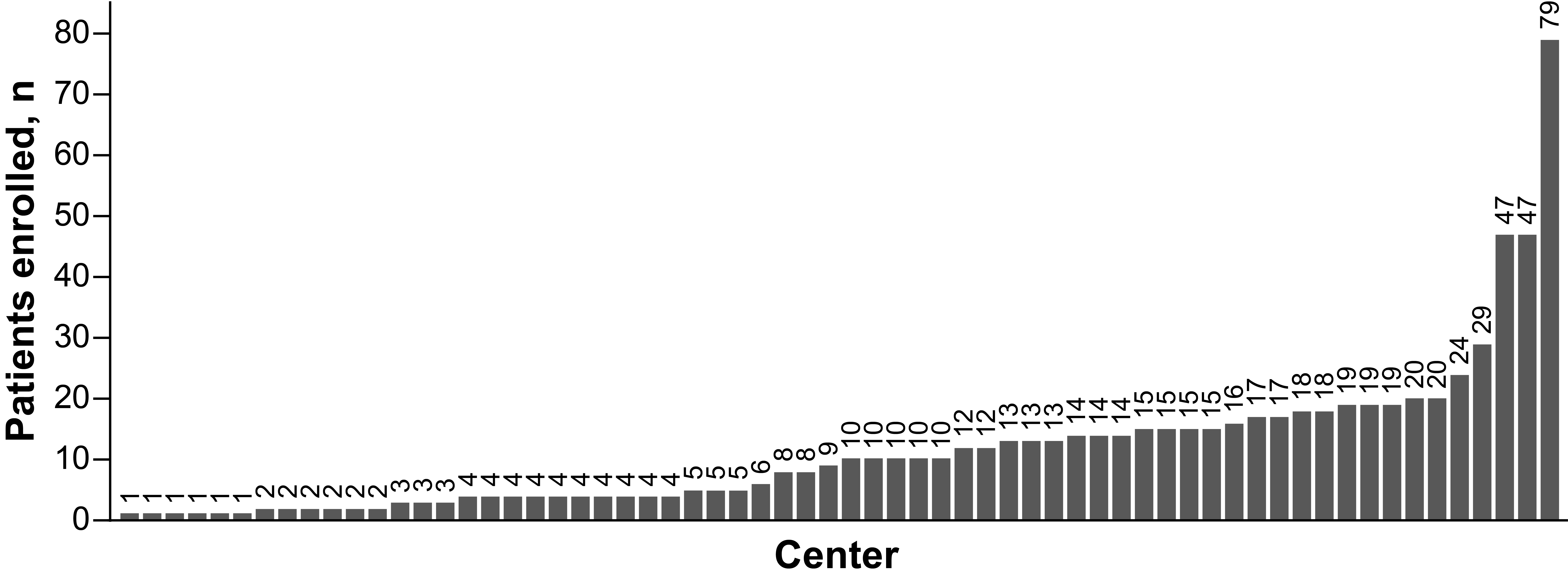

Supplement: Supplementary file 1 — Additional file 1: Figure S1. Number of patients in the analysis population of 737 patients enrolled at each of the 64 participating centers as of 30 June 2019. [file 12902_2021_888_MOESM1_ESM.zip › ID 77f8203a-9802-4232-ab46-9029d25bd44a_Additional File_Round 4.docx]
